# GAN-based synthetic brain PET image generation

**DOI:** 10.1186/s40708-020-00104-2

**Published:** 2020-03-30

**Authors:** Jyoti Islam, Yanqing Zhang

**Affiliations:** Department of Computer Science, Georgia State University, Atlanta, Georgia 30302-5060 USA

**Keywords:** Synthetic medical image generation, Positron emission tomography (PET), Generative adversarial networks, Brain imaging, Alzheimer’s disease

## Abstract

In recent days, deep learning technologies have achieved tremendous success in computer vision-related tasks with the help of large-scale annotated dataset. Obtaining such dataset for medical image analysis is very challenging. Working with the limited dataset and small amount of annotated samples makes it difficult to develop a robust automated disease diagnosis model. We propose a novel approach to generate synthetic medical images using generative adversarial networks (GANs). Our proposed model can create brain PET images for three different stages of Alzheimer’s disease—normal control (NC), mild cognitive impairment (MCI), and Alzheimer’s disease (AD).

## Introduction

**Fig. 1:**
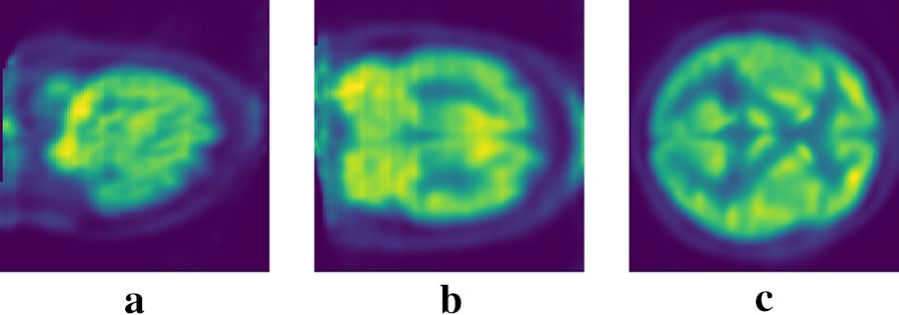
Example of brain PET images. **a** Sagittal view, **b** coronal view, **c** axial view

Developing AI-assisted automated disease diagnosis systems using medical images often requires a large training dataset with annotated samples, especially for supervised learning methods. Experts with good knowledge of the specific data and task are needed for performing such annotations. So, medical image annotation process is expensive in terms of time, money and effort. It becomes more challenging for precise annotations, such as for identifying different stages of Alzheimer’s disease. If diagnostic images are intended to be made public, patient consent may be necessary depending on the institutional protocols [1]. So there are very few public medical datasets available online, and they are still limited in size and quality. Collecting medical images for developing automated computer-aided diagnosis system is a complicated and expensive procedure and requires adequate funding, handling privacy concern, and collaboration of researchers, physicians, and hospitals. Medical datasets are often imbalanced as pathologic findings are usually rare, and it creates another challenge to train the automated diagnosis system (Fig. 1).

Data augmentation is one way to overcome the problem of limited dataset. There are several data augmentation techniques, such as translation, rotation, scale, flip, etc. But these techniques are not as useful for medical image analysis as they are for natural image dataset. On the contrary, techniques such as translation and rotation might change the pattern useful for the diagnosis. Besides, these images resemble a great extent to the original ones. So the ML model using these augmented data gain little performance improvements due to the lack of generalization abilities. Another type of data augmentation strategy is synthetic data generation. A synthetic dataset is generated programmatically. Such dataset is highly beneficial for medical image analysis. There is no patient data handling or privacy concerns as the data are produced synthetically. The dataset can contain samples from both positive and negative classes for diagnosis purpose and help build a generalized model.

Generating synthetic images for building a large-scale dataset for training deep learning model is an active research area. Image-to-image translation methods have made it possible to create such synthetic images. Image-to-image translation refers to the problem of translating a representation of an image into another, for example, converting an RGB image to BW image or vice versa. Both supervised and unsupervised technologies have been used for image-to-image translation. Generative Adversarial Networks (GANs) [2] can generate synthetic data with good generalization ability. GAN has two different networks—Generator and Discriminator. The model is trained in an adversarial process where the Generator generates fake images, and the Discriminator learns to discriminate between the real and fake images. Computer vision community have widely used GAN for image-to-image translation and done some excellent research works to generate synthetic data [3–10]. Such papers have achieved impressive results on background removal, palette generation, sketch to portrait, pose transfer, semantic segmentation [3], super resolution [4], style transfer [5], image inpainting [6], future state prediction [8], and image manipulation guided by user constraints [9]. The success of the vision community for synthetic data generation using GAN and the limitation of medical data inspired us to explore methods suitable for medical image synthesis. In this study, we focus on synthetic brain positron emission iomography (PET) image generation for different stages of Alzheimer’s disease—normal control (NC), mild cognitive impairment (MCI), and Alzheimer’s disease (AD).

Alzheimer’s disease (AD) is a severe neurological disorder and the most common type of dementia. The prevalence of Alzheimer’s disease is approximated to be around 5% after 65 years. In developed countries, the prevalence of Alzheimer’s disease is staggering 30% for more than 85 years old. There is a high probability that around 0.64 billion people will be diagnosed with Alzheimer’s disease by 2050 [11]. Alzheimer’s disease is incurable. The effect of Alzheimer’s disease is losing memory, ability to continue day-to-day activities and performing mental functions. At the initial stage, Alzheimer’s disease affects the brain part controlling memory and language functionality. So, patients suffer from memory loss, confusion, and difficulty in speaking, reading or writing. Alzheimer’s disease patients tend to forget about their life history and often cannot recognize family members. Alzheimer’s disease patients have difficulties in daily activities such as combing the hair or brushing the teeth. As a result, patients with Alzheimer’s disease become anxious or aggressive. As they forgot things, they often wander away from home. In the advanced stage, the brain part controlling breathing and heart functionality get destroyed, and that causes death.

Since Alzheimer’s disease is incurable, it is crucial to detect patients at the MCI stage before the disease progresses further. Earlier diagnosis can help in proper treatment and prevent brain tissue damage. Alzheimer’s disease causes degeneration of brain cells. Such changes can be captured using different imaging modalities, e.g., structural and functional magnetic resonance imaging (sMRI, fMRI), positron emission tomography (PET), single photon emission computed tomography (SPECT), and diffusion tensor imaging (DTI) scans, etc. With the progression of Alzheimer’s disease, the volume of abnormal proteins (amyloid-$\beta$ [A$\beta$] and hyperphosphorylated tau) increases in the brain. The accumulation of these proteins causes gradual changes in the brain and leads to progressive synaptic, neuronal and axonal damage. There is a stereotypical pattern of these changes, including early medial temporal lobe (entorhinal cortex and hippocampus) involvement, followed by progressive neocortical damage [12]. These changes often occur years before the symptoms of Alzheimer’s disease appear. The toxic hyperphosphorylated tau and/or amyloid-$\beta$ [A$\beta$] seems to slowly erode the brain. Finally, amnestic symptoms start to develop when a clinical threshold is surpassed.

Hippocampus is a part of the brain that controls episodic and spatial memory. It is a small but vital organ that works as a relay structure between the brain and the body. Alzheimer’s disease shrinks the hippocampus and cerebral cortex of the brain and enlarges the ventricles [13]. If the hippocampus is shrunk, it causes cell loss and damage to synapses and neuron ends. So neurons cannot communicate anymore via synapses. As a result, brain regions related to remembering (short-term memory), thinking, planning, and judgment are affected [13]. The degenerated brain cells can be captured using positron emission tomography (PET) for measuring these progressive changes. We propose a novel model to generate synthetic brain position emission tomography (PET) images exploiting Generative Adversarial Networks for three stages of Alzheimer’s disease—normal control (NC), mild cognitive impairment (MCI), and Alzheimer’s disease (AD).

The rest of the paper is organized as follows. Section 2 discusses briefly about the related work on synthetic medical data generation. Section 3 presents the proposed model. Section 4 reports the experimental details and the results. Finally, in Sect. 5, we conclude the paper with our future research direction.

## Related work

The recent advances of deep learning technologies have brought numerous breakthroughs in machine learning research and reached to a stage in some tasks where they provide similar or better performance than human. Some examples are image classification [14], intelligent driving [15], smart cities [16], voice recognition [17], playing Go [18], medical imaging [19–25], visual sentiment analysis [26], etc. The reason behind this success is significantly dependent on the size and the quality of the dataset being used to train the deep learning model. The scale and quality of the labeled or annotated data determine the performance of the deep learning model. Large-scale annotated dataset is required for training the model to achieve superior model performance. If the training-labeled dataset is small, the model fails to provide a good generalized performance. But obtaining such labeled data is difficult and expensive as it requires close and seamless collaboration from the outstanding experts in the field.

To address the insufficiency of the training dataset, researchers have proposed several oversampling methods. Duplicating the samples from minority class in a imbalanced dataset and adding artificial noise was proposed by DeRouin et al. [27]. Synthetic minority oversampling technique (SMOTE) was proposed by Chawla et al. [28] to create a synthetic dataset with samples from the minority class. Han et al. [29] proposed a Borderline-SMOTE method, considering neighboring instances and the minority instances near the borderline. Sample data generation using the weighted distribution for minority class instances based on the level of difficulty to learn them was proposed by He et al. [30]. Barua et al. [31] proposed a majority weighted minority oversampling technique using Euclidian distance-based clustering method to generate synthetic minority class samples. Xie et al. [32] introduced an oversampling technique by mapping the training samples in a low-dimensional space, assigning weights, and using the local densities. Their method addressed the problem of dimensionality that affected earlier methods. Douzas and Bacao [33] introduced a self-organizing map-based method using artificial data points in high-dimensional space. These oversampling methods helped to achieve more samples for the minority class for imbalanced datasets.

Some traditional approaches have been proposed to address the small sample size problem. Zhou and Jiang [34] trained a neural network and then employed it to generate a new training set, known as the neural-ensemble-based C4.5. Li and Lin [35] determined the probability density function of the training samples and used it to generate new samples. Li and Fang [36] used group discovery and parametric equations of the hypersphere to propose a non-linear classification technique to generate samples for enlarging the training dataset. These traditional methods are limited in their ability to learn the inherent features of the samples.

Synthetic image generation methods can be classified into two major categories. The first category is the model-based approach where a model is formulated to observe the data and a dedicated engine renders the data. This approach has been used for increasing the training dataset of urban driving environment [37, 38], object detection [39], text segmentation [40], realistic digital brain-phantom generation [41], synthetic agar plate image generation [42]. Designing such specialized data generation engine requires accurate model and deep knowledge of the specific domain. The other category of synthetic image generation method is known as the learning-based approach. These methods can learn the intrinsic spatial variability of the training image dataset. The probability distribution of the real images in the training dataset is learned implicitly by the model, and new images are generated by mimicking the original samples. Generative Adversarial Network is a learning-based approach. For synthetic image generation, both supervised [3, 43, 44] and unsupervised [10, 45–47] approaches are being used. In supervised training, a set of pairs of corresponding images ${(s_i, t_i)}$ are used, where $s_i$ is an image of the source domain and $t_i$ is a corresponding image in the target domain. For example, Pix2Pix [3] utilizes supervised training using a conditional GAN that learns to generate the output image based on the corresponding input image. The Generator network follows an encoder–decoder structure. The input of the Generator is the image from a particular domain A, and it learns to generate images in a different domain B. The Discriminator examines these generated images based on the training images from domain A and their corresponding images in domain B, and learns to distinguish between real and fake images. Based on the feedback from the discriminator, the Generator learns to generate more realistic images.

Generative adversarial network (GAN) [2] brought a breakthrough in the synthetic data generation research area. It can learn the distribution of the real dataset and generate synthetic samples conforming to that distribution. GAN have been successfully applied in image generation, image inpainting [48], image captioning [49–51], object detection [52], semantic segmentation [53, 54], natural language processing [55, 56], speech enhancement [57], credit card fraud detection [58] and supervised learning with insufficient training data [59]. From the experiments and results of these studies, it is evident that GAN conforms to the distribution of the original data samples and can generate realistic synthetic data. These promising applications in different fields also emphasize that GAN is independent of the precise domain knowledge for generating synthetic data.

Medical image synthesis and Generative Adversarial Networks have got attention in recent years. Costa et al. [60] used a fully convolutional neural network to train on retinal vessel segmentation images and then applied GANs for generating synthetic retinal images. Dai et al. [61] used GANs for creating lung fields and heart segmentation images from chest X-ray images. Gou et al. [62] proposed a method to employ a GAN to generate and learn from synthetic eye images to improve eye detection accuracy. Shin et al. [63] utilized GANs for generating synthetic abnormal MRI images with brain tumors. Nie et al. [64] proposed an auto-context model for brain CT and MRI image refinement. Schlegl et al. [65] trained GANs for anomaly detection in retinal images. Frid-Adar et al. [66] applied GANs for synthesizing liver lesion ROIs to apply in liver lesion classification. Hu et al. [67] applied GANs to generate a MRI motion model. Mahapatra et al. [68] synthesized high-resolution retinal fundus images using Generative Adversarial Networks. Nie et al. [64] generated synthetic pelvic CT images using GANs. Liu et al. [69] synthesized HCC samples using an approach based on a generative adversarial network (GAN) combined with a deep neural network. Han et al. proposed [70] a two-step GAN-based DA to generate and refine brain magnetic resonance (MR) images with/without tumors separately. Andreini et al. [71] proposed a GAN-based approach for synthesizing high-quality retinal images, along with the corresponding semantic label.

In our previous research works, we had to handle the limited dataset problem for Alzheimer’s disease diagnosis. There is a gap in research work for synthesizing brain images for Alzheimer’s disease diagnosis. Besides, there are very few works done for PET image synthesis. To mitigate these gaps, we propose a novel model to generate synthetic brain positron emission tomography (PET) images exploiting Generative Adversarial Networks for three stages of Alzheimer’s disease—normal control (NC), mild cognitive impairment (MCI), and Alzheimer’s disease (AD).

## Methodology

### Data selection

For our proposed model, we have used 411 PET scans (98 AD, 105 NC, 208 MCI) of 479 patients. We collected the data from the Alzheimer’s Disease Neuroimaging Initiative (ADNI) database (adni.loni.usc.edu). Specifically we used ADNI1 baseline dataset for our model. The subjects were in the age range 55–92. The ADNI was launched in 2003 as a public–private partnership, led by principal investigator Michael W. Weiner, MD. The primary goal of ADNI has been to test whether serial magnetic resonance imaging (MRI), positron emission tomography (PET), other biological markers, and clinical and neuropsychological assessment can be combined to measure the progression of mild cognitive impairment (MCI) and early Alzheimer’s disease (AD). Up-to-date information related ADNI database can be found at http://www.adni-info.org [72].

### Generative Adversarial Networks

Generative Adversarial Networks (GANs) is a deep learning architecture that consisted of two models—a generative model G and a discriminative model D. The generative model captures the data distribution. The discriminative model estimates the probability that the sample is drawn from the training data rather than the generative model. The two models are simultaneously trained via an adversarial process. The architecture is inspired by game theory and corresponds to a minimax two-player game. The training procedure of G is to maximize the probability of D making a mistake [2].

Let the generator* G *($z, \theta _{x}$) is a differentiable function represented by a multilayer perceptron with parameters $\theta _{g}$ that depicts a mapping to the data space. To learn the generator’s distribution $\rho _{g}$ over the data space *x*, a prior $\rho _{z}$ is defined on random input noise variables *z*. The discriminator* D* ($x, \theta _{d}$) is also a neural network that gets a sample the real dataset or the generated synthetic dataset produced by G and outputs a single scalar value that the input data comes from the real training dataset. The training process focuses on the task that the discriminator D will maximize the probability of assigning correct labels to the training examples and generated samples from G. At the same time, G is trained to generate data samples similar to the real dataset so that D cannot differentiate them from actual data. Similar to game theory, the discriminator D and the generator G play a two-player mini–max game with following value function *V*(*G*, *D*):

1 $$\begin{aligned} \underset{G}{\min}\,\underset{D}{\max} \,V(D,G) = &\, \mathbb {E}_{x\sim \rho _{{\text{data}}(x)}} [\log D(x)] \\& +\mathbb {E}_{z\sim \rho _{{\text{data}}(z)}} [\log (1-D(z))], \end{aligned}$$

where *x* is the real data and *z* is the input random noise. $\rho _{\text{data}}$, $\rho _{z}$ represent the distribution of the real data and the input noise. *D*(*x*) represents the probability that *x* came from the real data while *G*(*z*) represents the mapping to synthesize the real data. The generator G is a deeper neural network and has more convolutional layers and nonlinearities. The noise vector *z* is upsampled while G learns the weights through backpropagation. At some point, the generator starts producing data that are classified as real by the discriminator.

### Deep Convolutional Generative Adversarial Networks (DCGANs)

Deep Convolutional Generative Adversarial Networks (DCGAN) [73] is a major improvement on the first GAN [2]. DCGAN can generate better quality images and have more stability during the training stage. In the synthetic image generation process using the DCGAN, there are two phases: a learning phase and a generation phase. In the training phase, the generator draws samples from an N-dimension normal distribution and works on this random input noise vector by successive upsampling operations, eventually generating an image from it. The discriminator attempts to distinguish between images drawn from the generator and images from the training set [73].

Two important features in DCGAN are BatchNorm ([74] for regulating the extracted feature scale, and LeakyRelu [75] for preventing dead gradients. DCGAN also replace all max pooling with convolutional stride and use transposed convolution for upsampling. It eliminates fully connected layers and uses batch normalization. DCGAN uses ReLU in the generator except for the output which uses Tanh and uses LeakyReLU in the discriminator.

### Proposed model

**Fig. 2:**
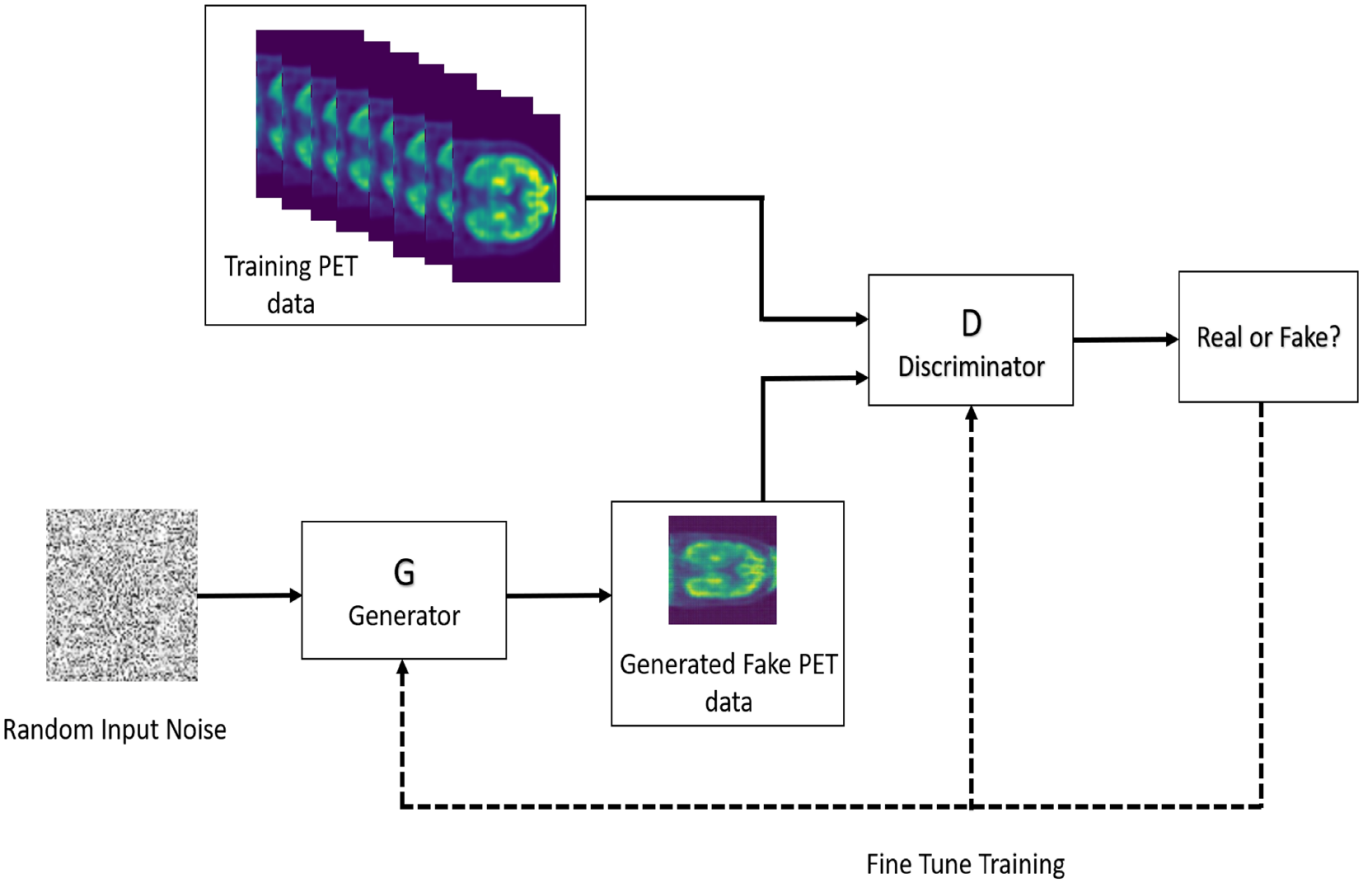
Proposed synthetic brain PET image generator

We propose a novel approach to produce synthetic PET images using a Deep Convolutional Generative Adversarial Networks. Following the guidelines to construct the generator and discriminator, described in the paper written by Radford et al. [73], we implemented and trained them on PET scan images using the original discriminator and generator cost functions. Figure 2 shows the proposed synthetic PET image generator model.

#### Generator architecture

**Fig. 3:**
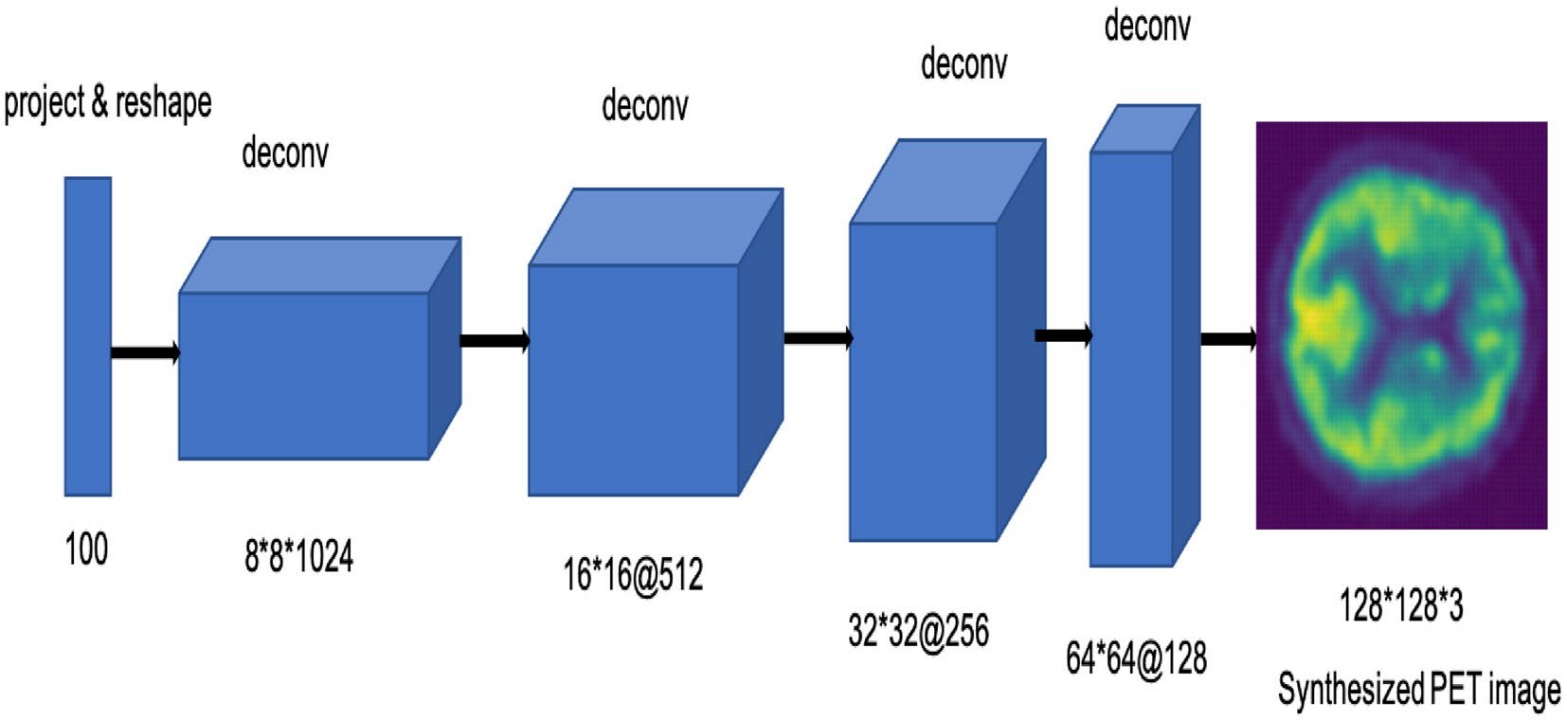
Generator architecture of the proposed model

**Fig. 4:**
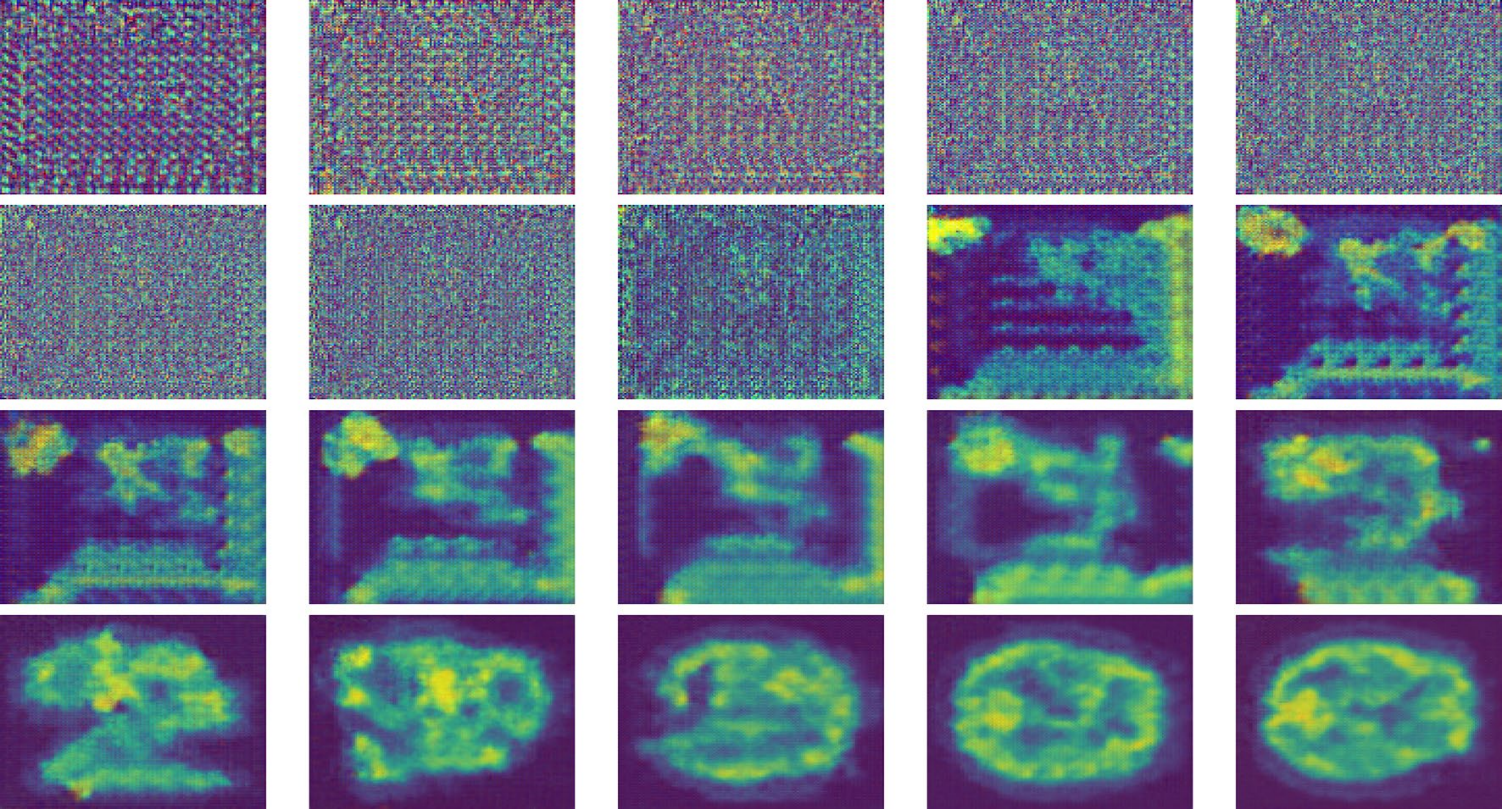
Visualization of the generator output in the training process

The input of the generator is a vector of random 100 numbers drawn from a uniform distribution, and the output is a brain PET image of size 128 * 128 * 3. The generator architecture is shown in Fig. 3. The network has a fully connected layer and five strided convolutional transpose (known also as ‘deconv’) layers. The strided convolutional transpose layers transform the latent vector into a volume with shape 128 * 128 * 3. Each convolutional transpose layer is paired with a 2d batch norm layer and a ReLU activation. The strided convolutional transpose layer inserts zeros in between the pixels of the input vector and expands it. The convolution operation is performed over the enlarged vector to create bigger output data. Normalizing responses to have zero mean and unit variance over the entire mini-batch are applied to stabilize the learning process. Figure 4 shows the output of different steps from the generator of the proposed model.

#### Discriminator architecture

**Fig. 5:**
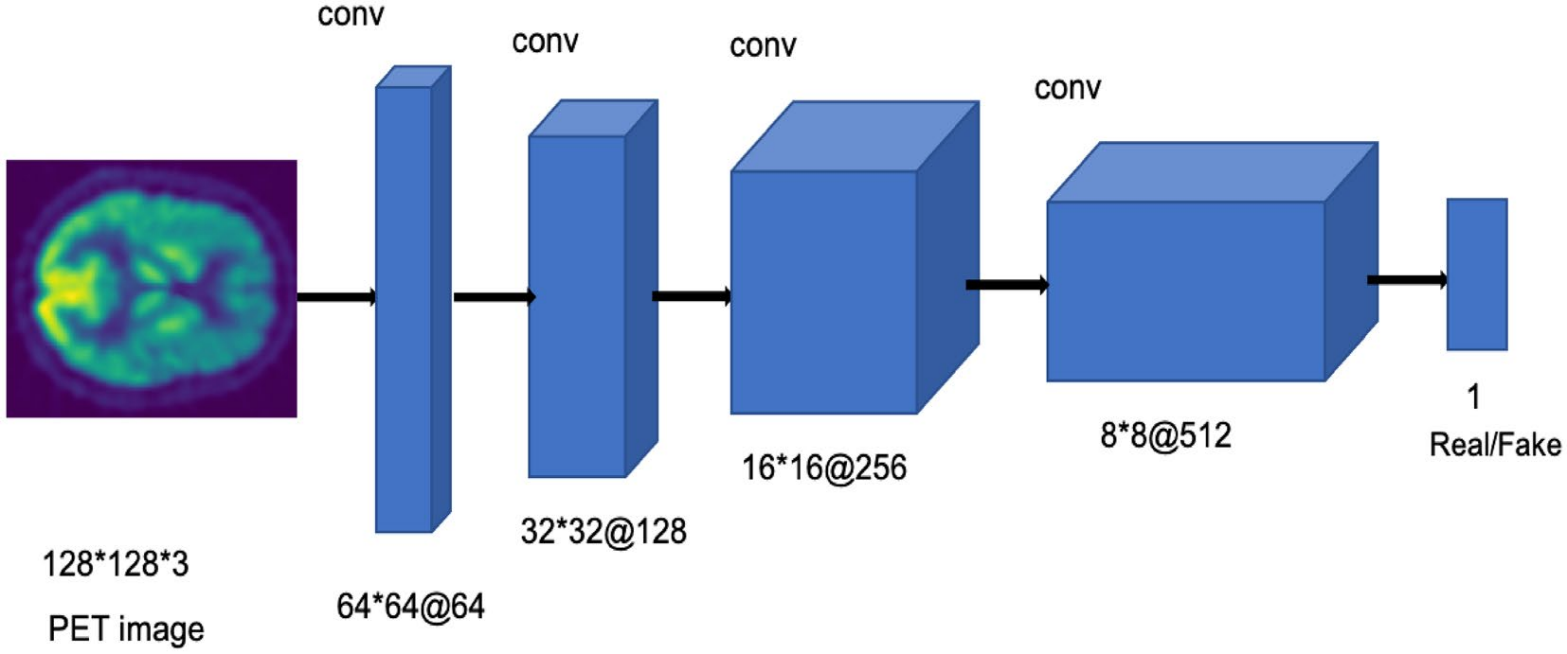
Discriminator architecture of the proposed model

The discriminator network consists of a CNN architecture that takes an image of size 128 * 128 * 3 (brain PET image) as input. The discriminator analyzes the input brain PET image and decides if it is real or fake. The network consists of five convolution layers with a kernel size of 5 * 5 and a fully connected layer. Strided convolutions are applied to each convolutional layer to reduce spatial dimensionality instead of using pooling layers. Batch-normalization and Leaky ReLU activation are applied to each convolutional layer of the network except the output layer. The fully connected output layer has a Sigmoid function to generate the likelihood probability (0,1) score of the input image to be real or fake. The discriminator architecture is shown in Fig. 5.

#### Training procedure

We trained the proposed model to synthesize brain PET images for three stages of Alzheimer’s disease separately. The training process was done iteratively for the generator and the discriminator. We used mini-batches of* m* = 64 brain PET examples for each stage (NC, MCI, and AD) and* m* = 64 noise samples drawn from a uniform distribution between [− 1, 1]. In the Leaky ReLU, the slope of the leak was set to leak = 0.2. We initialized the weights to a zero-centered normal distribution with a standard deviation of 0.02. Stochastic gradient descent was used in the training process with the Adam optimizer, an adaptive moment estimation that incorporates the first and second moments of the gradients, controlled by parameters $\beta _{1} = 0.$5 and $\beta _{2} = 0.999,$ respectively. We applied a learning rate of 0.0001 for 500 epochs.

In the training process, the discriminator is trained to maximize the probability of assigning correct labels to the training examples and the generated samples. At first, the discriminator gets a batch of real samples from the training set. The batch is forward passed through D, and the loss (*log*(*D*(*x*))) is calculated. The gradients are calculated in a backward pass. Then, a batch of fake samples from the generator is forward passed through D. Similarly, the loss $(\log(1-D(G(z))))$ is calculated, and the gradients are accumulated with a backward pass. Finally, the gradients from both the all-real and all-fake batches are summed up, and a step of the Discriminator’s optimizer is done.

The Generator is trained to generate better fake samples by minimizing $\log(1-D(G(z)))$. The training process maximizes *log*(*D*(*G*(*z*))) to minimize the generator’s loss $\log(1-D(G(z)))$. The output of the generator is passed to the discriminator, and the classification result is collected. The training process repeats unless the generator learns to generate samples labeled as real by the Discriminator.

## Experiments and results

It is an open issue to develop objective metrics that correlate with perceived quality measurement. For quality evaluation of synthetic images, it should be specific for each application. Following previous state-of-the-art, we performed a quantitative and qualitative assessment of our proposed model. To our best knowledge, no previous works attempted to generate synthetic brain PET images using real PET images. We quantitatively compare the predicted results in terms of peak signal to noise ratio (PSNR) and structural similarity Iniex (SSIM). PSNR is used to measure the ratio between the maximum possible intensity value and the mean squared error of the synthetic and the real image:

2 $$\begin{aligned} {\text{PSNR}} = 10 \log_{10}\frac{(\max(y))^{2} }{^{\frac{1}{n}\sum _{i}^{n} (y_{i}-\hat{y_{i}})^2}}, \end{aligned}$$

where* n* is the number of pixels in an image. For our proposed model, the mean PSNR of real and generated images is 32.83.

Structural similarity index (SSIM) finds the similarities within pixels of two image; i.e., if the pixels in the two images line up and or have similar pixel density values:

3 $$\begin{aligned} (x,y) = \frac{(2\mu _x\mu _y + C_1) + (2 \sigma _{xy} + C_2)}{(\mu _x^2 + \mu _y^2+C_1) (\sigma _x^2 + \sigma _y^2+C_2)}, \end{aligned}$$

where *x* is the estimated PET and *y* is the ground truth PET, $\mu _x$ is the average of *x*, $\mu _y$ is the average of *y*, $\mu _x^2$ is the variance of *x*, $\mu _y^2$ is the variance of *y*, $\sigma _{xy}$ is the covariance of *x* and *y*. $C_1 = (k_{1}L)^2$ and $C_2 = (k_{2}L)^2$ are used to stabilize the division with weak denominator, where *L* is the dynamic range of the pixel-values, $k_1= 0.01$ and $k_2= 0.03$. For our proposed model, the mean SSIM of real and generated images is 77.48.

**Fig. 6:**
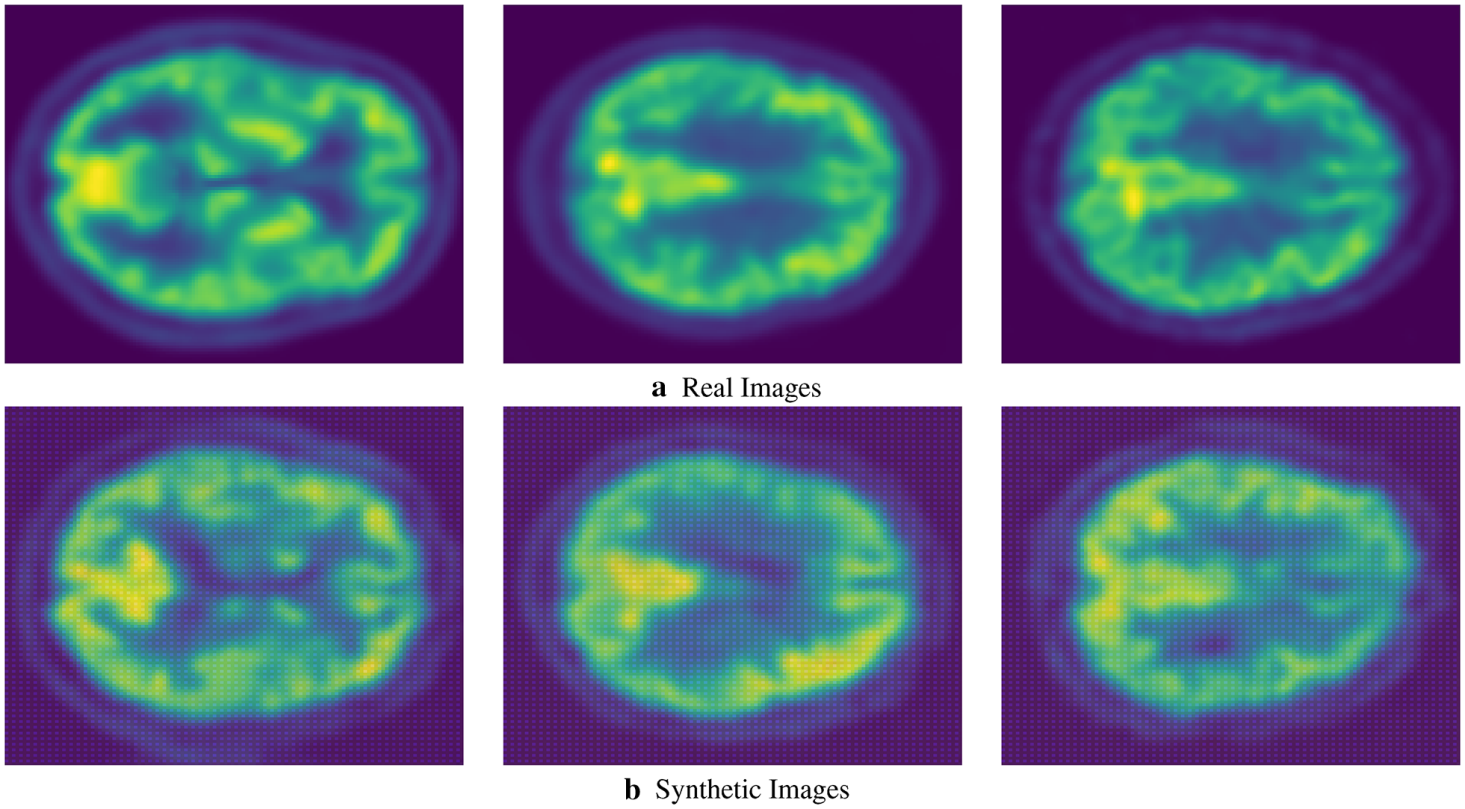
Real and synthetic brain PET images of normal patient: **a** real **b** synthetic

**Fig. 7:**
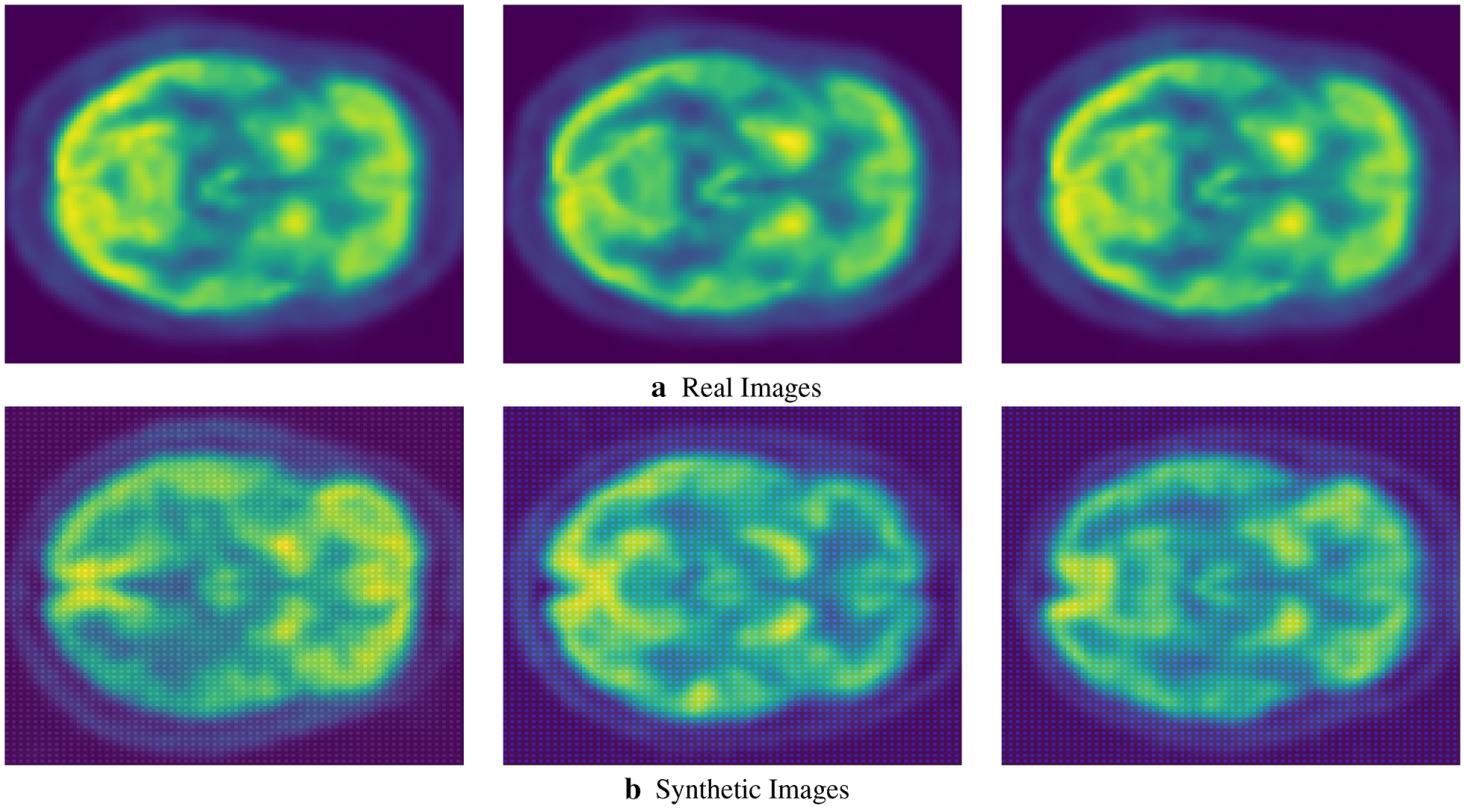
Real and synthetic brain PET images of MCI patient: **a** real **b** synthetic

**Fig. 8:**
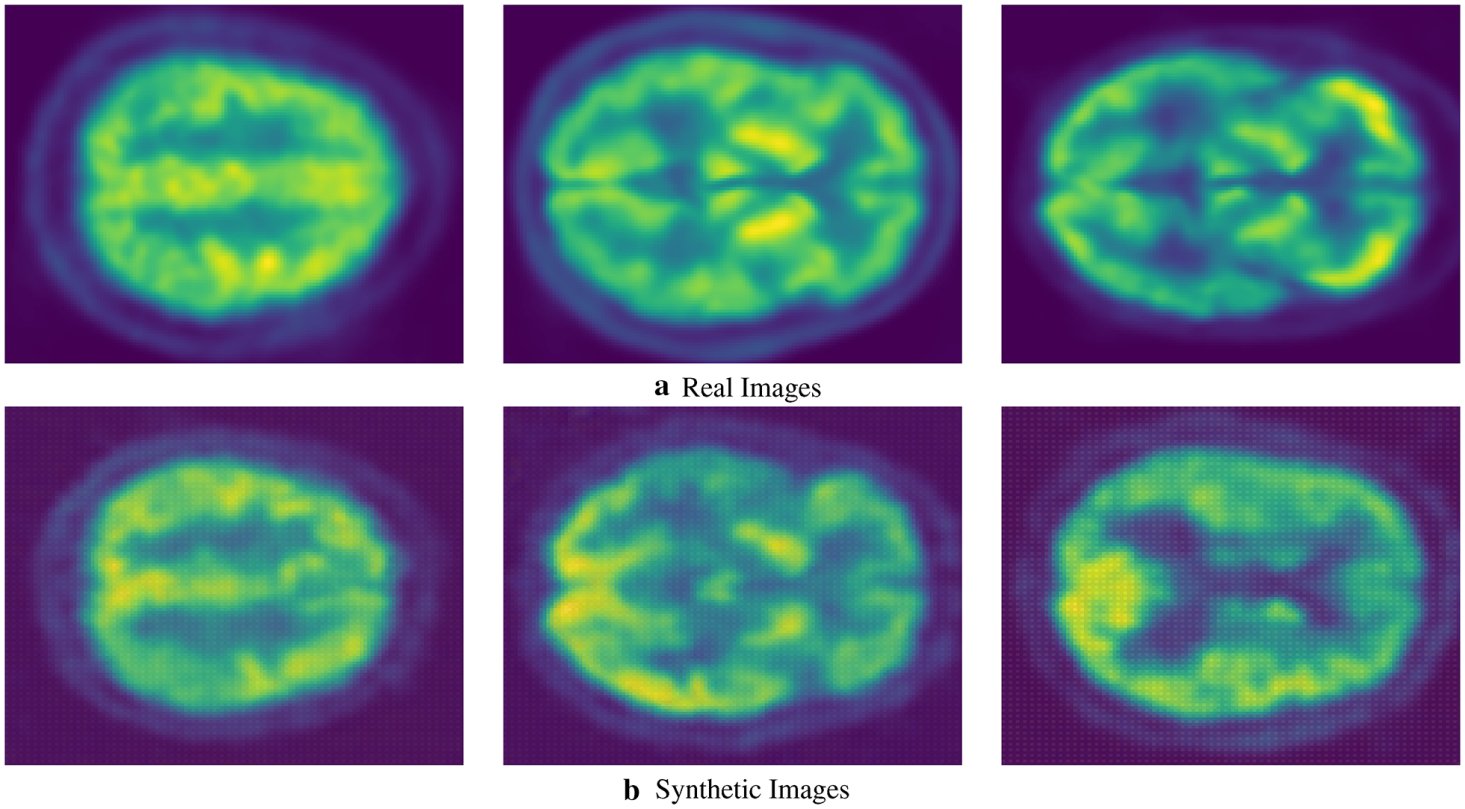
Real and synthetic brain PET images of AD patient: **a** real **b** synthetic

**Fig. 9:**
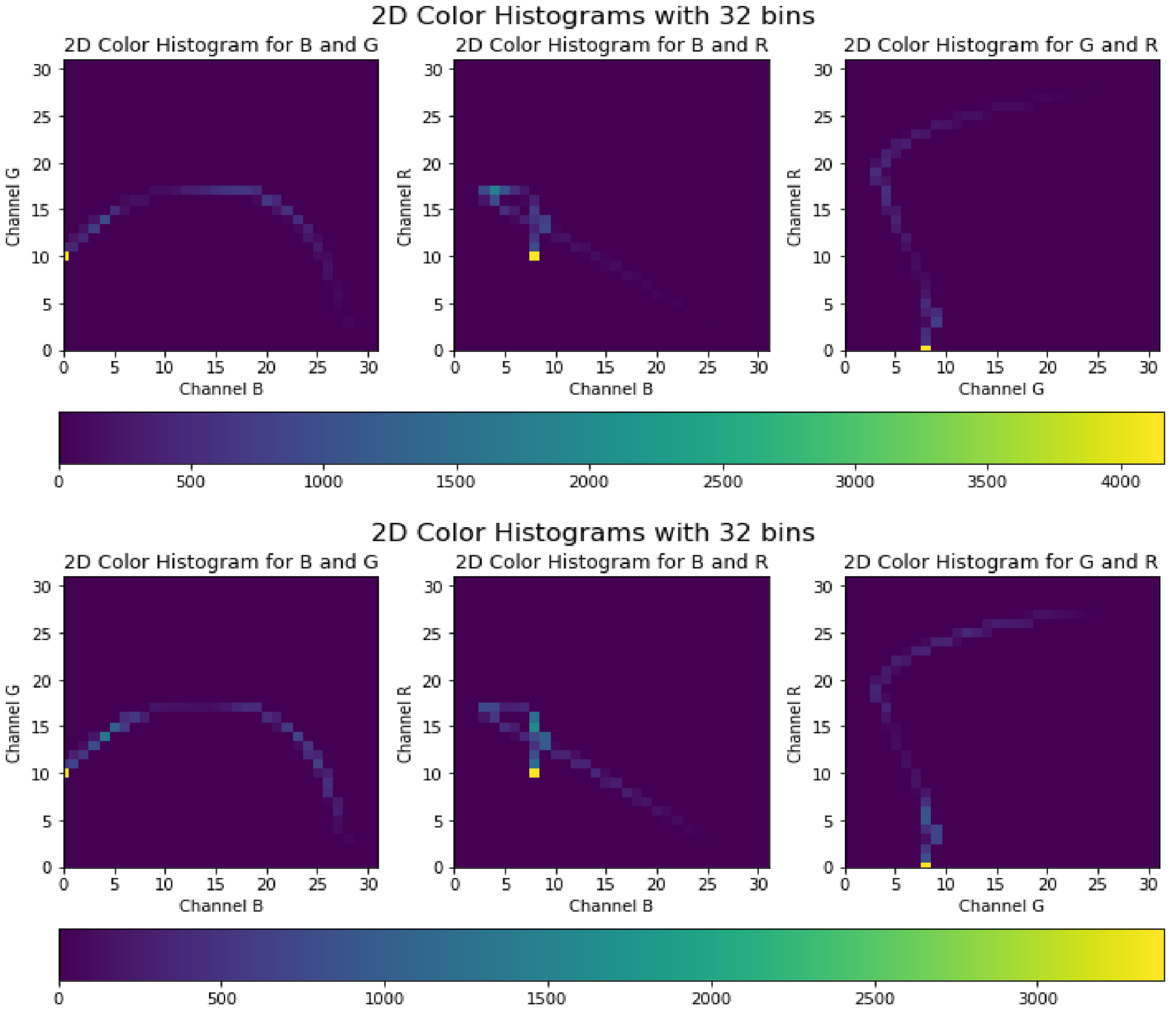
2D-histograms of the synthetic and real images. **a** 2D-histogram of real images, **b** 2D-histogram of synthetic images

We present sample visual results of representative slices from the generated PET data for qualitative comparison. Figures 6,  7, and  8 show the synthesized PET images from NC, MCI, and AD patients, respectively. From the results, we could see that the synthesized brain PET images are quite similar to the real brain PET images. To analyze the similarity between synthetic and real images, we also obtained the 2D-histogram of real and synthetic images. Figure 9 presents the 2D-histogram of a sample real and synthetic image [76]. We also developed a 2D-CNN model using axial, coronal, and sagittal slices from the generated PET data. The model achieved 71.45% classification accuracy for CN/AD classification, that is 10% more than the classification model trained without the generated synthetic data.

## Conclusions

We conclude that synthetic medical image generation is a promising research area and cost-saving approach for developing automated diagnostic technology. Our proposed model can be generalized in other disease diagnosis systems using PET images and can help to supplement the training dataset. The qualitative and quantitative evaluation of the proposed model demonstrates that the synthesized images are close to real brain PET images of different stages of Alzheimer’s disease. We believe that our proposed model can help to generate labeled images and aid data augmentation for developing robust disease diagnosis systems, and eventually save lives. There are several limitations to the proposed work. One possible extension could be an increase from 2-D to 3-D input volumes, using 3-D GAN, at the cost of a longer processing time and an increased memory usage. We trained separate GANs for each stage of Alzheimer’s disease, which increased the training complexity. Future research can focus on the investigation of GAN architectures that generate multi-class samples together.

## Data Availability

The dataset used for this research work is publicly available at http://www.adni-info.org.
